# Type Disparity in Sodium–Glucose Cotransporter-2 Inhibitors in Incidences of Renal Cell Carcinoma: A Propensity-Score-Matched Cohort Study

**DOI:** 10.3390/cancers16112145

**Published:** 2024-06-05

**Authors:** Tsung-Kun Lin, Wei-Yao Wang, Tsung-Yuan Yang, Gwo-Ping Jong

**Affiliations:** 1 Department of Pharmacy, Tri-Service General Hospital, Taipei 114202, Taiwan; ray1007@gmail.com; 2 School of Pharmacy, National Defense Medical Center, Taipei 114201, Taiwan; 3 Department of Internal Medicine, Chung Shan Medical University Hospital, Taichung 40201, Taiwan; wywang1966@ndmctsgh.edu.tw; 4 Institute of Medicine, Chung Shan Medical University, Taichung 40201, Taiwan

**Keywords:** renal cell carcinoma, SGLT2 inhibitors, type 2 DM, type disparity

## Abstract

**Simple Summary:**

Sodium–glucose cotransporter-2 inhibitors (SGLT2Is) have been reported to be associated with renal cell carcinoma (RCC) risk. However, the effect between individual SGLT2Is on RCC incidence in patients with type 2 diabetes (T2D) is unclear. In this study, we aimed to explore type disparity in the prescription of SGLT2Is on RCC risk. After a 5.5-year follow-up, SGLT2I use was associated with a significantly lower risk of incident RCC. Furthermore, significant disparity was observed in SGLT2I use, with lower rates observed in dapagliflozin and empagliflozin, in contrast to canagliflozin.

**Abstract:**

(1) Background: Recently, sodium–glucose cotransporter-2 inhibitors (SGLT2Is) have been reported to significantly reduce renal cell carcinoma (RCC) risk. However, the effect between individual SGLT2Is on RCC incidence in patients with type 2 diabetes (T2D) or heart failure is unclear. We conducted an observational analysis to explore type disparity in the prescription of SGLT2Is on RCC risk. (2) Methods: A nationwide retrospective cohort study using the Health and Welfare Data Science Center database (2016–2021) was conducted. Patients aged ≥40 years who took SGLT2Is were designated as the SGLT2I group, whereas propensity score 1:1-matched randomly selected patients without SGLT2Is were assigned to the non-SGLT2I group. The primary outcome was the risk of incident RCC between individual SGLT2Is. Multiple Cox regression modeling was conducted to analyze the association between individual SGLT2I use and RCC risk. (3) Results: After a 5.5-year follow-up, SGLT2I use was associated with a significantly lower risk of incident RCC (hazard: 0.62; 95% confidence interval [CI]: 0.44–0.89). Compared with non-users and after adjusting for the index year, sex, age, comorbidities, concurrent medication, and the risk of developing RCC, the hazard ratios of dapagliflozin, canagliflozin, and empagliflozin were 0.66 (95% CI: 0.53–0.83), 0.84 (95% CI: 0.46–1.30), and 0.71 (95% CI: 0.56–0.90), respectively. (4) Conclusions: Our data show a type-based effect of SGLT2Is on RCC risk. The type-based effect of SGLT2Is should be further studied for better clinical management information and for reducing RCC incidence in patients with T2D.

## 1. Introduction

In the past decade, diabetes and RCC incidence has increased worldwide, particularly in developing countries [1,2]. Diabetes is a risk factor for RCC, and poor outcomes have been observed in patients with type 2 diabetes (T2D) and comorbidity with RCC [1,2,3]. T2D and RCC have become a significant health burden and a concern in every country [4]. Therefore, decreasing the incidence of RCC in patients with T2D is crucial.

Sodium–glucose cotransporter-2 inhibitors (SGLT2Is) are a group of antidiabetic and heart failure drugs that target the SGLT2 protein and have been shown to reduce the risk of major adverse cardiovascular events in patients with diabetes who are at a high cardiovascular risk of heart failure and prevent or reduce kidney function failure [5,6,7,8]. Recently, several in vitro cell culture studies have found that SGLT2Is may exhibit antiproliferative activity against tumor cells and reduce the risk of cancer [9,10,11]. Kuang et al. demonstrated how dapagliflozin may play a critical role in regulating the cell cycle and apoptosis. Additionally, it reduces glucose uptake and inhibits tumor growth in vitro and in vivo [12]. However, Phillips et al. reported that empagliflozin increased renal tumor growth in male mice in the high-dose group [9]. However, no in vitro and in vivo studies have investigated the use of canagliflozin in RCC patients [13,14]. These findings highlight the need to investigate associations between type disparity in SGLT2Is and RCC in patients with T2D. Therefore, this study aimed to identify type disparity in SGLT2Is in a real-world cohort of patients with T2D from the National Health Insurance Research Database (NHIRD).

## 2. Materials and Methods

### 2.1. Study Design

This retrospective cohort study examined national clinical and administrative data using the NHIRD from 2016 to 2021. The NHIRD used for this study is representative of the Taiwanese population and includes twenty-three million people [15]. The NHIRD was the primary source for claims data of National Health Insurance beneficiaries, which included prescription data, diagnostic codes, inpatient claims, outpatient claims, registry for medical facilities, and registry for board-certified specialists. Our study was approved by the Ethics Committee of the Chung Shan Medical University Hospital (CS2-22032), and written consent was waived because the NHIRD de-identifies and encrypts the names of patients, health care providers, and medical institutions.

### 2.2. Study Population

This study included adults with T2D as measured by the International Classification of Diseases, 10th Revision, Clinical Modification (ICD-10-CM) code E11 or 150, who were treated with an SGLT2I on admission to the hospital or as outpatients between May 2016 and December 2021.

The study group (SGLT2I users) consisted of patients who received at least one SGLT2I prescription for more than 180 days during the study period. The control group (non-SGLT2I users) consisted of randomly selected T2D patients who did not receive any SGLT2I prescription throughout the study period. The inclusion criteria were (1) those aged ≥40 years, (2) those who had two or more outpatient visits and continuously received an SGLTI for more than six months during the study period, or (3) those who had one or more admissions with a diagnosis of T2D or HF and continuously received an SGLTI for more than six months. Comorbidities related to T2D or HF were recorded according to the ICD-10-CM code and included coronary heart disease (ICD-10-CM code I20–I25), hypertension (ICD-10-CM code I10), hyperlipidemia (ICD-9-CM code E78.1–E78.5), chronic liver disease (ICD-10-CM code K71, K75, K76), stroke (ICD-10-CM codes I60, I61, I62, I63, I65, I66, I67.84, G45, G46), chronic obstructive pulmonary disease (ICD-10-CM code J44), atrial fibrillation and flutter (ICD-10-CM code I48), and rheumatoid arthritis (ICD-9-CM code M05). Exclusion criteria included (1) those having a history of RCC before the index date, (2) those whose follow-up was less than 6 months, and (3) those less than 40 years old. To account for the differences in baseline characteristics and RCC risk between the SGLT2I users and the control group, the groups were matched by a propensity score at a ratio of 1:1. The index date was defined as the first SGLT2I prescription between May 2016 and December 2021.

### 2.3. Variables and Outcomes

The baseline characteristics, assessed on the index date, included gender, age, T2D duration, comorbidities, and concurrent medication. Comorbidities and medication use were restricted to prescriptions given more than 6 months before the index date. The study endpoint was the development of RCC, defined as the first occurrence of the RCC code (ICD-10-CM codes C64) in inpatient or outpatient claim records during follow-up until December 2021.

### 2.4. Statistical Analysis

The study group taking SGLT2 inhibitors as antidiabetic drugs was matched 1:1 by age, sex, comorbidity, current medication, and propensity score to the group not taking SGLT2 inhibitors (control group). Differences in baseline patient characteristics were examined using the absolute standardized differences (ASDs) between SGLT2I users and non-users. An ASD mean difference of >0.1 indicated a significant difference in potential confounders between the two groups. In both cohorts, the incidence rates of RCC were calculated as per 10,000 person-months. A time-dependent Cox proportional hazards regression model was used to compare the RCC risk between the two groups. Hazard ratios and 95% confidence intervals were calculated after adjusting for age, sex, index date, concurrent medications, and comorbidities for RCC risk. All statistical calculations were performed using statistical analysis software, version 9.3 (SAS Institute, Inc., Cary, NC, USA).

## 3. Results

### 3.1. Study Population

**Figure 1:**
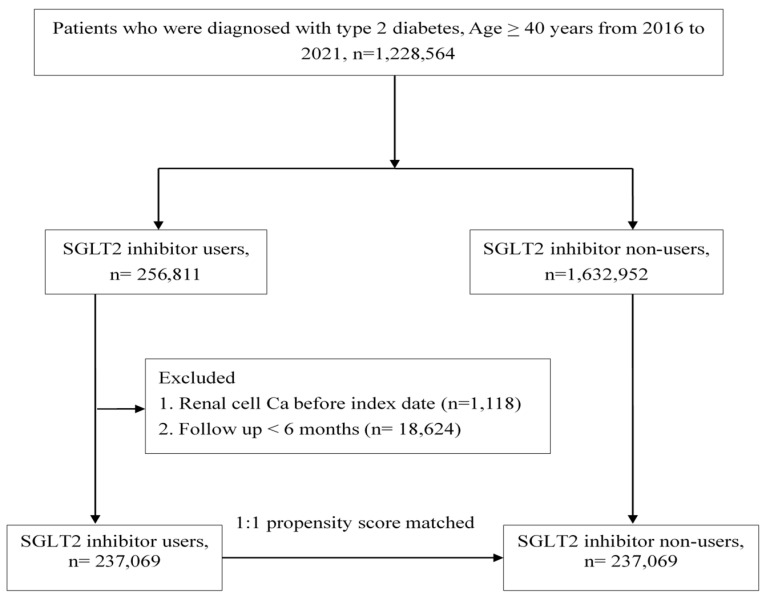
Flowchart of study population.

Overall, 256,811 T2D or heart failure patients who received their first SGLT2I prescriptions between May 2016 and December 2021 were retrospectively studied. Finally, after exclusions, 237,069 individuals in the study group and 237,069 control patients matched by propensity score at a 1:1 ratio were included (Figure 1). The SGLT2I group patients had more comorbidities at baseline (except for hypertension and chronic kidney disease) and used more concurrent medication (except for aspirin) than the control group patients (Table 1).

### 3.2. Relative Risk of RCC in Individual SGLT2Is

**Figure 2:**
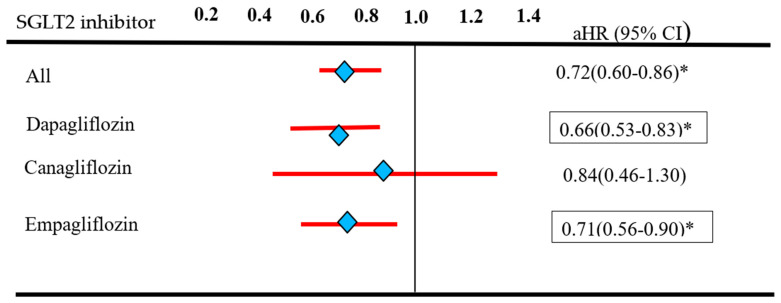
Relative Risk of RCC in individual SGLT2 inhibitors. * *p* < 0.05.

The crude incidence rate of RCC was 0.38 per 10,000 person-months (95% CI: 0.32–0.45) for SGLT2I users compared with 0.56 (95% CI: 0.50–0.64) for non-SGLT2I users. A significantly lower RCC incidence was noted in the SGLT2I group than in the control group (crude HR: 0.71; 95% CI: 0.59–0.85) (Table 2). The result is consistent after adjustments for the index date, sex, age, comorbidities, and concurrent medication at baseline (adjusted HR: 0.72; 95% CI: 0.60–0.86). Regarding the effects of individual SGLT2I treatment on RCC incidence after adjusting for potential confounders, dapagliflozin had 0.66 HR (95% CI 0.53–0.83), canagliflozin had 0.84 HR (95% CI 0.46–1.30), and empagliflozin had 0.71 HR (95% CI 0.56–0.90) than in the non-SGLT2I group (Figure 2).

## 4. Discussion

This study showed that SGLT2I use in patients with T2D decreased RCC risk. However, significant type disparity was observed in SGLT2I use, with lower rates observed in dapagliflozin and empagliflozin, in contrast to canagliflozin.

The use of sodium–glucose cotransporter-2 (SGLT2) inhibitors is a safe and efficacious approach to managing type 2 diabetes and heart failure [16,17,18,19]. Recently, SGLT2Is have been shown to have anticancer effects. In vitro and in vivo studies have confirmed that SGLT2Is exhibit an antiproliferative activity against some types of tumors in cell cultures [10,20]. The anticancer activity of SGLT2Is has been demonstrated in breast, liver, pancreatic, prostate, bowel, and lung cancer [21]. However, these effects are under-studied in patients with diabetes who are at a higher risk of aggressive RCC, especially regarding type disparity in SGLT2Is. A recent meta-analysis revealed that SGLT2Is significantly increased RCC risk, especially with the potential effects of dapagliflozin and ertugliflozin, although not reaching statistical significance [22]. However, another meta-analysis showed that SGLT2Is did not significantly increase RCC risk [23]. Simultaneously, a Scandinavian cohort study reported that SGLT2Is did not significantly increase RCC risk compared to GLP-1 receptor agonists and reduced RCC risk instead [24]. Moreover, this study revealed that the use of SGLT2Is in patients with T2D decreased RCC risk. The mechanism of SGLT2Is in anticancer therapy may interfere with the utilization of glucose by tumors, the induction of apoptosis, the inhibition of angiogenesis, and reduction in inflammation [10].

Previous in vitro studies demonstrated that empagliflozin inhibits oxidative DNA damage, mutagenesis, and tumor growth in CD-1 female mice and rats, but it did not do so in male mice and rats [8]. Additionally, dapagliflozin use was associated with reduced RCC risk in vitro and in vivo studies [10,11]. However, no study has examined the association between canagliflozin and RCC in vitro and in vivo [12,13]. These data show a type-based effect of SGLT2Is on RCC risk. This study highlights the type-based effect of SGLT2Is on RCC risk. The differences in association by type are not fully understood. They may indicate different etiological pathways for individual SGLT2I effects on RCC risk. Further prospective studies of the type-based effect of SGLT2Is are warranted for better clinical management information and for reducing RCC incidence in patients with T2D.

Interestingly, this study showed that canagliflozin was not significantly associated with decreased RCC risk. SGLT1 was found to be overexpressed in the gastrointestinal tract, kidney, and heart, whereas SGLT2 is highly selectively expressed in the kidney [25,26]. Canagliflozin demonstrated potent SGLT1 inhibitory activity compared with dapagliflozin and empagliflozin [26]. This might be partially explained in our results. A study reported that the duration of drug prescription may be a risk factor for RCC [27]. Dapagliflozin and empagliflozin were prescribed in May 2016, whereas, in this dataset, canagliflozin was prescribed in March 2018. The relatively short duration of canagliflozin prescription compared with those of dapagliflozin and empagliflozin may be a major risk factor for RCC. Thus, long-term prospective studies are needed to clarify our findings.

The strengths of our study include our use of a population-based database and a large number of RCC case subjects compared with other published studies. Furthermore, our findings used propensity score matching to control for potential confounders, which made our hypothesis feasible. Our study is the first cohort study to provide evidence of type disparity for an association between the use of SGLT2Is on RCC risk in patients with T2D. Our findings support the hypothesis that type disparity in SGLT2I prescription in patients with T2D is associated with the risk of RCC.

Our study had several limitations. First, we could not directly assess the severity of T2D and/or glucose control and did not have reliable information on the number of types of medications used. Second, the underlying diagnosis, outcome, and comorbidities in the NHIRD, registered by each physician, may have been miscoded or misclassified. However, because the data were population-based, we assumed that there were no differences between the groups. Third, the laboratory data such as hemoglobin A1c levels, fasting blood sugar levels, liver function, renal function, and thyroid function were not available from the claims data. Therefore, our findings may not be generalizable to patients in other countries. Further prospective randomized control trials are needed.

## 5. Conclusions

We conclude that there is a type-based effect of SGLT2Is on RCC risk. The type-based effect of SGLT2Is should be further studied for better clinical management information and for reducing RCC incidence in patients with T2D. Further efforts are necessary to maximize the potential population benefit of individual types of SGLTI therapy in high-risk groups.

## Figures and Tables

**Table 1:** Patient and clinical characteristics.

	Non-SGLT2I N = 237,069	SGLT2I N = 237,069	ASD
Sex			0.0045
Female	102,366 (43.18%)	103,718 (43.75%)	
Male	134,703 (56.82%)	133,351 (56.25%)	
Age			0.0000
<50	52,511 (22.15%)	52,511 (22.15%)	
50–59	67,446 (28.45%)	67,375 (28.42%)	
60–69	76,170 (32.13%)	76,241 (32.16%)	
≥70	40,942 (17.27%)	40,942 (17.27%)	
Comorbidity			
Hypertension	147,220 (62.10%)	145,181 (61.24%)	0.0157
CAD	38,974 (16.44%)	39,496 (16.66%)	0.0060
Hyperlipidemia	160,733 (67.80%)	158,101 (66.69%)	0.0236
Chronic kidney disease	66,047 (27.86%)	63,961 (26.98%)	0.0221
Liver disease	31,720 (13.38%)	31,886 (13.45%)	0.0000
Stroke	12,470 (5.26%)	12,517 (5.28%)	0.0008
COPD	11,806 (4.98%)	12,043 (5.08%)	0.0042
Atrial fibrillation and flutter	3461 (1.46%)	3509 (1.48%)	0.0013
Rheumatoid Arthritis	1754 (0.74%)	1802 (0.76%)	0.0016
Medication			
NSAIDs	164,881 (69.55%)	165,427 (69.78%)	0.0051
Corticosteroids	59,860 (25.25%)	60,547 (25.54%)	0.0066
PPIs	20,672 (8.72%)	20,933 (8.83%)	0.0037
H2 blockers	78,517 (33.12%)	78,991 (33.32%)	0.0045
Aspirin	67,446 (28.45%)	67,138 (28.32%)	0.0023
Statin	165,735 (69.91%)	165,806 (69.94%)	0.0213
Biguanides	152,720 (64.42%)	152,791 (64.45%)	0.0010
Sulfonylureas	107,345 (45.28%)	108,435 (45.74%)	0.0110
Alpha glucosidase inhibitors	44,071 (18.59%)	45,565 (19.22%)	0.0163
Thiazolidinediones	41,297 (17.42%)	42,269 (17.83%)	0.0108
DPP4 inhibitors	93,524 (39.45%)	93,642 (39.50%)	0.0031
Insullin	47,177 (19.90%)	47,935 (20.22%)	0.0081
GLP-1 agonists	4575 (1.93%)	4623 (1.95%)	0.0053
SGLT2I type			
Non-SGLT2I	237,069 (100%)	0 (0%)	
Dapagliflozin	0 (0%)	117,397 (49.52%)	
Canagliflozin	0 (0%)	21,881 (9.23%)	
Empagliflozin	0 (0%)	97,791 (41.25%)	

SGLT2I: Sodium–glucose cotransporter-2 inhibitor; ASD: absolute standardized difference; CAD: coronary artery disease; COPD: chronic obstructive pulmonary disease; NSAIDs: non-steroidal anti-inflammatory drugs; PPIs: proton pump inhibitors; DPP4: Dipeptidyl peptidase-4; GLP-1: Glucagon-like peptide-1.

**Table 2:** Incidence rate in study groups.

	Non-SGLT2I	SGLT2I	*p*-Value
N	237,069	237,069	
Follow-up person-months	5,684,030	5,688,388	
New case	318	216	
Incidence rate * (95% CI)	0.56 (0.50–0.64)	0.38 (0.32–0.45)	
Crude Relative risk (95% CI)	reference	0.71 (0.59–0.85)	<0.0001
Adjusted HR (95% CI) †	reference	0.72 (0.60–0.86)	<0.0001

* Incidence rate per 10,000 person-months. † adjusted hazard ratio (HR), the covariates including year of index, sex, age, co-morbidities, and medication at baseline. SGLT2I: Sodium–glucose cotransporter-2 inhibitor; CI: confidence interval.

## Data Availability

The data used during this current study are available from the corresponding authors on reasonable request.
